# Assessing Uveitis Risk following Pediatric Down Syndrome Diagnosis: A TriNetX Database Study

**DOI:** 10.3390/medicina60050710

**Published:** 2024-04-25

**Authors:** Alan Y. Hsu, Yu-Hsun Wang, Chun-Ju Lin, You-Ling Li, Ning-Yi Hsia, Chun-Ting Lai, Hou-Ting Kuo, Huan-Sheng Chen, Yi-Yu Tsai, James Cheng-Chung Wei

**Affiliations:** 1 Department of Ophthalmology, China Medical University Hospital, China Medical University, Taichung 40202, Taiwan; alanhsu1221@gmail.com (A.Y.H.); doctoraga@gmail.com (C.-J.L.); withwind037@yahoo.com.tw (C.-T.L.); a0934305326@gmail.com (H.-T.K.); yiyutsai@seed.net.tw (Y.-Y.T.); 2 Department of Medical Research, Chung Shan Medical University Hospital, Taichung 40201, Taiwan; cshe731@csh.org.tw (Y.-H.W.); jccwei@gmail.com (J.C.-C.W.); 3 School of Medicine, College of Medicine, China Medical University, Taichung 40402, Taiwan; 4 Department of Optometry, Asia University, Taichung 40447, Taiwan; 5 Department of General Medicine, China Medical University Hospital, Taichung 40201, Taiwan; 6 An-Shin Dialysis Center, NephroCare Ltd., Fresenius Medical Care, Taichung 43655, Taiwan; dalusoha@gmail.com; 7 Institute of Medicine, Chung Shan Medical University, Taichung 40201, Taiwan; 8 Department of Allergy, Immunology & Rheumatology, Chung Shan Medical University Hospital, Taichung 40201, Taiwan; 9 Institute of Integrated Medicine, China Medical University, Taichung 40402, Taiwan

**Keywords:** Down syndrome, TriNetX, retrospective, uveitis, pediatric

## Abstract

*Background and Objectives:* The risks of uveitis development among pediatric patients with Down syndrome (DS) remain unclear. Therefore, we aimed to determine the risk of uveitis following a diagnosis of DS. *Materials and Methods:* This multi-institutional retrospective cohort study utilized the TriNetX database to identify individuals aged 18 years and younger with and without a diagnosis of DS between 1 January 2000 and 31 December 2023. The non-DS cohort consisted of randomly selected control patients matched by selected variables. This included gender, age, ethnicity, and certain comorbidities. The main outcome is the incidence of new-onset uveitis. Statistical analysis of the uveitis risk was reported using hazard ratios (HRs) and 95% confidence intervals (CIs). Separate analyses of the uveitis risk among DS patients based on age groups and gender were also performed. *Results:* A total of 53,993 individuals with DS (46.83% female, 58.26% white, mean age at index 5.21 ± 5.76 years) and 53,993 non-DS individuals (45.56% female, 58.28% white, mean age at index 5.21 ± 5.76 years) were recruited from the TriNetX database. Our analysis also showed no overall increased risk of uveitis among DS patients (HR: 1.33 [CI: 0.89–1.99]) compared to the non-DS cohort across the 23-year study period. Subgroup analyses based on different age groups showed that those aged 0–1 year (HR: 1.36 [CI: 0.68–2.72]), 0–5 years (HR: 1.34 [CI: 0.75–2.39]), and 6–18 years (HR: 1.15 [CI: 0.67–1.96]) were found to have no association with uveitis risk compared to their respective non-DS comparators. There was also no increased risk of uveitis among females (HR: 1.49 [CI: 0.87–2.56]) or males (HR: 0.82 [CI: 0.48–1.41]) with DS compared to their respective non-DS comparators. *Conclusions:* Our study found no overall increased risk of uveitis following a diagnosis of DS compared to a matched control population.

## 1. Introduction

Down syndrome (DS) is a congenital condition caused by aberrations in the chromosomal 21 area and is known to affect approximately 1 in 700 births in the United States. Data from Asian countries like Taiwan and Japan reveal that the live birth rate of infants with DS is around 7.79 per 100,000 live births and 9.7 per 100,000 live births, respectively [1,2]. These epidemiological figures associated with DS underscore its importance in global public health. DS has been linked with various comorbidities, including arthropathy, autoimmune diseases, and congenital heart disease. Ophthalmologic disorders among DS individuals have also been described and encompass high refractive errors, eyelid abnormalities, and congenital cataracts [3]. However, the connection between DS and uveitis, an inflammatory condition affecting the uveal tract of the eye, has not been well established. This is notable, considering that DS is associated with a heightened proinflammatory state, characterized by elevated circulating interleukin levels, and has been correlated with other inflammatory conditions like arthropathy [4,5]. Immune dysregulation is central to the manifestation of uveitis, a condition involving inflammation of the uveal tract. Uveitis can be broadly categorized based on anatomical locations into anterior, intermediate, and posterior uveitis. Uveitis has been documented in other conditions with similar pathophysiological mechanisms, such as juvenile idiopathic arthritis [6]. Hence, it is probable that uveitis could manifest in individuals with DS, with inflammation serving as a key pathological link between the two conditions. Three small case series [7,8] have reported such an association between uveitis and Down syndrome. The case series from Zaki et al. is one example, where they reported anterior and intermediate uveitis among the DS patients they recruited [7]. A better understanding of the potential associations between uveitis and Down syndrome (DS) is important for the optimal clinical management of such patients. The often asymptomatic nature of uveitis among pediatric patients, along with issues related to noncompliance with examinations among patients of such an age, can impede the timely diagnosis and management of intraocular inflammatory disorders like uveitis among DS patients. If the results of our study can definitively demonstrate an increased risk of intraocular inflammatory disorders such as uveitis occurring among pediatric patients with Down syndrome, clinicians caring for these patients should adopt increased clinical vigilance concerning uveitis occurrence. The incidence of uveitis among pediatric patients is relatively uncommon compared to uveitis of adult-onset, as one study reported that pediatric uveitis accounts for only 2% of all cases. The majority of uveitis begins around the ages of 35 to 45 years of age [9]. When studying diseases with such a low incidence rate, being able to recruit a large sample size can aid in determining the presence or absence of associations between conditions. The largest case series on this topic only involved 190 DS patients [10]. Comparatively, the TriNetX platform, a federated multicenter database encompassing extensive health records of over a hundred million patients throughout the United States, is ideal for conducting comprehensive epidemiological studies on rare conditions such as pediatric uveitis, and the use of this platform has been validated in previous investigations [11].

Therefore, we aimed to explore the potential association between uveitis and DS by conducting the largest population-based study using the multinational TriNetX electronic registry platform. 

## 2. Materials and Methods

Our retrospective, multinational study used the TriNetX platform, a cloud-based clinical information registry that stores millions of deidentified patient data. The data include basic demographics and diagnosis (via the International Classification of Diseases, Tenth Revision, Clinical Modification, ICD-10-CM codes). 

In terms of privacy protection, the TriNetX database adheres to the highest standard set by the ISO 27001:2013 and Section §164,514(b)(1) of the Health Insurance Portability and Accountability Act (HIPPA) [12]. Based on compliance with well-established privacy standards, the China Medical University Hospital Institutional Review Board waived the patient consent requirement. The study followed the Strengthening the Reporting of Observational Studies in Epidemiology (STROBE) reporting guidelines and the Declaration of Helsinki. 

### 2.1. Study Participants, Main Measures and Outcomes 

Supplementary Figures S1 and S2 depict our cohort construction. All patients aged 18 years or younger diagnosed with DS from 1 January 2000 to 31 December 2023 were recruited and designated as our DS cohort. We set the index event for the DS cohort as a first-time encounter diagnosis based on the ICD-10 diagnostic code designation (refer to Supplementary Table S1).

We assigned those without DS diagnosis as the non-DS comparison cohort (refer to Supplementary Figure S2). The non-DS cohort was constructed from a randomized sampling of individuals from the TriNetX database who did not meet our inclusion criteria. The index date of the non-DS cohort was based on a randomized date derived from the last healthcare visit with an ophthalmologist between 2000 and 2023.

We also excluded certain conditions like viral hepatitis and human deficiency virus (HIV) to control for confounding effects at baseline (refer to Supplementary Table S1). 

Demographics (e.g., age, gender, and ethnicity)-related clinical information, including comorbidities, were extracted up to one year before the index date. Propensity score matching (PSM) was also utilized as part of our study design to ensure the equal distribution of baseline variables. Using such PSM, a 1:1 cohort that was made of DS and non-DS individuals was constructed for our study. The variables considered as part of our PSM included comorbidities of interest, gender, age, and ethnicity. This matching process was accomplished through the TriNetX electronic platform’s already-built function. 

The primary endpoint for our study was designated as the new-onset encounter diagnosis ICD-10 code of uveitis (refer to Supplementary Table S1) after the index date. We selected these diagnostic codes for uveitis, because they represent distinct anatomical subclasses. For instance, iridocyclitis encompasses anterior uveitis, while chorioretinal inflammation and retinal vasculitis pertain to posterior uveitis. The use of such diagnostic codes, as well as the other aspects of our study design, has been validated in previous studies [11,13]. 

### 2.2. Statistical Analysis 

All statistical analyses were conducted using the built-in functions of the TriNetX platform. We used the standardized mean differences (SMDs) to assess the distribution balance among our baseline variables. Variables with SMD values of less than 0.1 were considered well matched. The uveitis risk across different variables within the matched cohort was evaluated using Cox proportional hazards regression analysis. Hazard ratios (HRs) and 95% confidence intervals (CIs) were reported. The incidence of uveitis among DS patients was determined using the log-rank test and the Kaplan–Meier method. Statistical significance was defined as a two-sided *p*-value < 0.05.

## 3. Results 

### 3.1. Baseline Characteristics 

Our study included 53,993 individuals with DS and 53,993 non-DS individuals (refer to Table 1). After propensity score matching (PSM), our Down syndrome (DS) cohort demonstrated a gender distribution of 46.83% female and 52.61% male. Regarding ethnicity, 58.26% were of White descent, 11.13% were Black, and 3.06% were Asian. The mean age at index was 5.21 years, with a standard deviation of 5.76 for the DS cohort. Regarding comorbidities, 2.23% of DS patients had hypothyroidism, 1.47% had asthma, 0.81% had congenital heart malformations, 0.59% had hypertensive diseases, 0.28% had atopic dermatitis, 0.13% had celiac disease, 0.06% had Crohn’s disease, and 0.03% had ulcerative colitis. In comparison, our non-DS cohort post-PSM had a gender distribution of 45.56% female and 52.60% male. Regarding ethnicity, 58.27% were White, 11.12% were Black, and 3.06% were Asian. The mean age at index was 5.21 years, with a standard deviation of 5.76. Regarding comorbidities, 2.24% of non-DS patients had hypothyroidism, 1.45% had asthma, 0.80% had congenital heart malformations, 0.59% had hypertensive diseases, 0.27% had atopic dermatitis, 0.12% had celiac disease, 0.07% had Crohn’s disease, and 0.03% had ulcerative colitis. Baseline clinical information, obtained at the one-year time point before the index date from both cohorts (DS and non-DS), included demographics (e.g., age, gender, and ethnicity) and comorbidities (e.g., asthma, congenital malformation of the heart, and hypertensive diseases). This clinical information was collected both before and after PSM. SMD values of less than 0.1 were observed across most variables, indicating well-distributed characteristics.

### 3.2. Risk of Uveitis among Down’s Syndrome Individuals 

In our analysis of the risk of uveitis-related diseases among individuals with DS, we observed no overall association with uveitis-related diseases when comparing the DS cohort to the non-DS cohort (HR: 1.33 [CI: 0.89–1.99]). Additionally, specific conditions such as iridocyclitis (HR: 1.28 [CI: 0.83–1.99]), chorioretinal inflammation (HR: 1.42 [CI: 0.51–4.01]), and sympathetic uveitis (HR: 3.28 [CI: 0.36–29.54]) showed no statistical association with the DS cohort compared to the non-DS cohort across the entire study duration (refer to Table 2).

**Figure 1:**
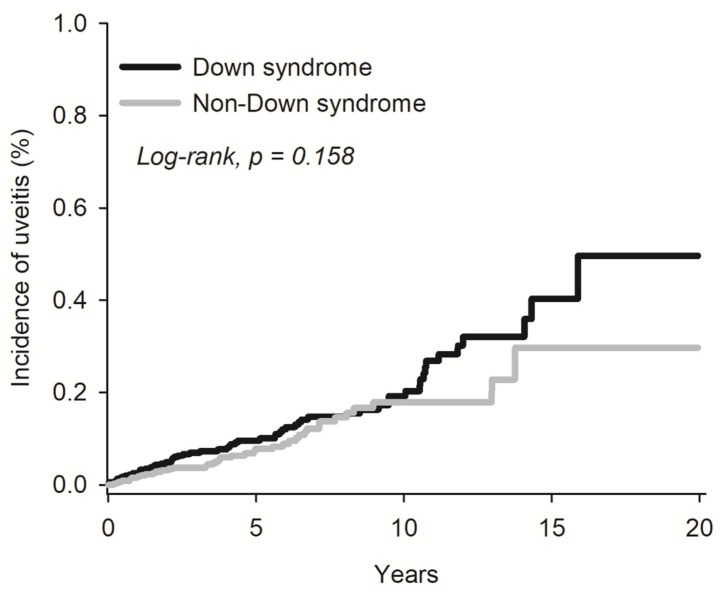
Kaplan–Meier analysis for the risk of uveitis.

Furthermore, Kaplan–Meier survival statistics and the associated log-rank test did not reveal any increased risk of uveitis among DS patients compared to the non-DS cohort (log-rank test, *p* = 0.158) (refer to Figure 1).

### 3.3. Stratification of Uveitis Risk Based on Age and Gender among Down’s Syndrome Individuals 

In our analysis of the risk of uveitis-related diseases among individuals with DS based on age (refer to Table 3), we observed no increased risk for uveitis within the age groups of 0–1 year (HR: 1.36 [CI: 0.68–2.72]), 0–5 years (HR: 1.34 [CI: 0.75–2.39]), and 6–18 years (HR: 1.15 [CI: 0.67–1.96]) compared to their respective non-DS comparators.

Similarly, in our analysis of the risk of uveitis-related diseases among DS patients based on gender (refer to Table 3), we found no increased risk for uveitis among females (HR: 1.49 [CI: 0.87–2.56]) and males (HR: 0.82 [CI: 0.48–1.41]) compared to their respective non-DS comparators.

## 4. Discussion

### 4.1. Novel Findings 

Our findings indicated no statistically significant association with uveitis risk among pediatric patients with DS compared to the non-DS cohort over the entire study period. Additionally, our stratification analysis based on age groups (0–1 year old, 0–5 years old, and 6–18 years old) and gender did not reveal any increased risk of uveitis among the DS cohort compared to their non-DS comparators.

### 4.2. Clinical Implications

Our study represents the first and largest investigation to date exploring the potential association between uveitis and DS using a multinational electronic health records database. Our study holds significant clinical implications, as identifying any potential associations could inform guidelines for the care of individuals with DS. The current guidelines, particularly those from the United States, predominantly emphasize clinical awareness for common ophthalmologic disorders observed in DS patients, such as nystagmus, nasolacrimal duct occlusion, and decreased visual acuity secondary to high refractive errors [14]. Notably, these guidelines do not mention uveitis, despite reports of its occurrence in three case series involving DS patients [7,8]. Should an association between uveitis and DS exist, it would be imperative to emphasize the clinical awareness of this ophthalmologic condition among healthcare providers. While we did not find evidence supporting an association between uveitis and DS, the implications of our findings remain significant. Our study suggests that if there is any association between uveitis and DS, it may not be as strong as initially suspected. This finding has significant implications, especially in pediatric ophthalmology settings, as it provides reassurance that the risk of uveitis among the eyes of DS pediatric patients is not strong. Missing a uveitis diagnosis among pediatric eyes can have devastating consequences for visual outcomes. However, despite the danger of a missed uveitis diagnosis, capturing uveitis in pediatric eyes can be challenging, particularly in DS-affected children. Ensuring compliance with ophthalmological examinations in this population can be difficult, further complicating the diagnostic process. This challenge is particularly pronounced when attempting to diagnose mild cases of uveitis among pediatric DS patients [15]. Therefore, our results are relevant for pediatric ophthalmologists and clinicians, suggesting that clinical efforts should be directed towards diagnosing other ophthalmological disorders more commonly observed in this population.

### 4.3. Comparison to Other Studies 

Only three case series have reported an association between uveitis and DS. Zaki et al.’s case series reported an association between uveitis and DS. In their study, which included eight DS subjects, all individuals exhibited bilateral uveitis, with a mean age of 29 years and a predominance of females (six out of eight cases) [7]. Most cases from Zaki et al. presented with anterior and intermediate uveitis. In contrast, our study employed a retrospective, multinational approach utilizing electronic health records to recruit 53,993 DS patients and compare them to 53,993 propensity-matched controls to assess their uveitis risk. Our study population had a mean age of 5.21 years, with females comprising 46.83%. Comparatively, we did not observe an overall association with uveitis risk or an increased risk among diagnostic codes specific to anterior uveitis. Due to the lack of specificity in the available electronic diagnostic codes for intermediate uveitis, we are unable to draw any definitive conclusions regarding the risk of intermediate uveitis among Down syndrome (DS) patients compared to the non-DS cohort [13].

Regarding gender, Zaki et al.’s study population consisted predominantly of females (six out eight patients), while our study had a male predominance. This difference should be noted, as females have been demonstrated to be more predisposed as autoimmune and toward non-infectious uveitis development. Such a predisposition has been linked to the stronger T-helper 2 response and hormone-mediated modulation seen in women [16]. However, despite the difference in gender makeup between our study and Zaki et al.’s, such an effect from gender differences should theoretically be modest due to the high power of our study (consisting of 53,933 DS individuals) and our study’s PSM that accounted for gender.

Zaki et al. noted that the mean onset of uveitis in DS patients occurred in the third decade of life. This observation regarding an older age for uveitis onset among DS patients was also correlated with another population-based study, where the mean age at first uveitis diagnosis was 10 years old for idiopathic uveitis among pediatric patients [17]. It is plausible that our study’s focus on a younger age group may have contributed to the underestimation of uveitis risk among DS patients.

Furthermore, Zaki et al. observed that three out of the eight DS patients with uveitis developed antiretinal antibodies, raising questions about whether certain subtypes of DS patients, particularly those with higher autoantibody levels or genetic or HLA-related differences, may predispose such a sub-cohort of DS patients to develop autoimmune-related conditions like uveitis. Such questions are difficult to answer with electronic diagnostic code studies, and future studies employing laboratory serum investigations are needed to confirm our findings.

In another case series by Mathan et al., only one case of uveitis was identified among 190 DS patients, yielding a prevalence of 0.53% [10]. Additionally, Mathan et al. observed that a percentage of their DS study population exhibited autoimmune conditions, with 12.63% having thyroid dysfunction, 6.84% diagnosed with celiac disease, and 3.16% presenting with psoriasis. Notably, eight DS patients from Mathan et al. had two or more comorbid autoimmune conditions. While these findings partially align with our own, indicating a weak association between uveitis and DS individuals, the potential of uveitis occurrence among DS cannot be entirely dismissed. Moreover, the presence of comorbid autoimmune conditions among some DS patients in the Mathan et al. study further underscores the potential autoimmune links between DS and uveitis. Other studies also support this notion, demonstrating elevated levels of inflammatory and anti-inflammatory cytokines in children with DS compared to age-matched controls [18]. Such an inflammatory predisposition may cause DS individuals to be more likely to develop inflammatory eye conditions like uveitis. However, due to the limitations of electronic diagnostic codes, we cannot ascertain how the inflammatory status of our DS patients impacted our study results.

In a separate study conducted by Liza-Sharmini et al. [8], they examined 60 patients with Down syndrome, with a mean age of 6.72 years and a female predominance of 56.7%. Their findings indicated that 1.7% of their total cases developed chronic uveitis. Although there were notable differences in the study population compared to ours, particularly in terms of gender and ethnicity, the mean age of their study population was closer to ours. Their results once again suggest a weak association between uveitis and Down syndrome. Notably, most of their study population comprised a specific ethnicity of Malay origin, accounting for 93%, whereas our study predominantly included individuals of White ethnicity. It is possible that ethnicity could have influenced the incidence of certain subtypes of uveitis, which may have impacted our results [19]. Therefore, further studies are needed to confirm our findings. 

Our study also yielded an intriguing finding concerning the low numbers of retinal vasculitis or panuveitis from among both our DS and non-Down syndrome (DS) patients. This is noteworthy, given the epidemiological data from previous studies regarding retinal vasculitis and panuveitis among pediatric patients. For instance, a study in Switzerland reported 5.1% of pediatric patients with panuveitis [20]. Moreover, the annual incidence of retinal vasculitis has been estimated to be around 1–2 per 10,000 individuals [21]. These epidemiological data suggest that our large study cohort of DS and non-DS patients should have captured at least some retinal vasculitis or panuveitis cases. The low number of retinal vasculitis or panuveitis recruited from our database may hint at the challenges clinicians face when diagnosing such ophthalmological conditions among pediatric patients. As previously mentioned, factors such as poor compliance with examinations and the often asymptomatic nature of uveitis among pediatric patients could contribute to this absence of findings. 

## 5. Strength and Limitations

The strength of our study lies in several key aspects. Firstly, it represents the first and largest investigation into the association between uveitis and DS, filling a critical gap in the existing literature. Moreover, our study design incorporates methodologies such as PSM and stringent exclusion criteria to increase the reliability of our results. Lastly, the extended follow-up period of 23 years provides valuable insights into any potential long-term association between uveitis and DS.

However, our study has several limitations that warrant consideration. One significant limitation is the reliance on electronic diagnostic codes, which may not accurately capture all relevant clinical information. For instance, factors like HLA gene typing, inflammatory serum markers, and the presence of autoantibodies are not typically recorded in diagnostic codes and, therefore, could not be accounted for in our analysis. The absence of such detailed clinical data may have influenced our ability to fully assess the impact of these factors on the risk of uveitis among DS patients. 

Another limitation related to diagnostic codes is the absence of specific codes for intermediate uveitis. Intermediate uveitis, defined by the SUN working group, is where inflammation is located in the vitreous [22]. However, as highlighted in a multicenter survey by Mckay et al., most electronic health records and related studies utilizing such records often erroneously categorize intermediate uveitis with the ICD-10-CM code for iridocyclitis, which, according to the SUN working definition, falls under the anatomical subtype for anterior-located uveitis [13]. Such limitations inherent in the existing diagnostic codes restrict our ability to evaluate the risk of intermediate uveitis among DS patients. 

Furthermore, we could not exclude the influence of medication history on our results. Given that leukemia and other cancer diagnoses have been reported among DS patients, treatment for such conditions often includes the use of steroids and other immunomodulatory agents like methotrexate. The use of such medications could have influenced the risk of future development of uveitis among these patients and potentially impacted our results as well. Future studies should perhaps account for such factors in order to better delineate the association between uveitis and DS diagnosis. 

One notable limitation pertains to ethnicity within our study. The ethnic distribution from our study population was primarily comprised of White individuals, with relatively lower numbers of Blacks and Asians. The disparity may have arisen from broader issues with healthcare access, as individuals of White ethnicity are more likely to have easier access to healthcare compared to those of other ethnicities [23]. Consequently, the limited representation of Blacks and Asians in our study restricts the generalizability of our findings, particularly to regions or countries with predominant African or Asian populations. To address these limitations and enhance the generalizability of uveitis risk assessments among DS patients, future studies should aim for greater ethnic diversity representation.

## 6. Conclusions and Future Directions 

Our study is the first and largest multi-institutional study to explore the association between uveitis and DS. Our findings revealed no statistically significant relationship between uveitis and pediatric patients with DS compared to non-DS patients. The results of our study hold significant implications for the clinical management of pediatric patients with Down syndrome (DS). However, our findings are constrained by several limitations, some of which are mentioned earlier. These limitations include the imbalanced representation of ethnicities in our study sample, the absence of data on antiretinal antibodies or other inflammatory-related laboratory results, and the difficulty in diagnosing uveitis from among patients of pediatric age. To address these gaps and achieve a more comprehensive understanding of the relationship between uveitis and DS patients, future studies could explore several avenues. Firstly, future studies can aim to conduct multicenter prospective investigations involving countries with more diverse ethnic compositions. Such a study design would greatly improve the generalizability of any future findings. Furthermore, incorporating laboratory results such as leukocyte counts, C-reactive protein (CRP) levels, and antiretinal antibodies could provide deeper insights into the relationship between uveitis and DS. Previous case series have highlighted the presence of antiretinal antibodies among DS patients with uveitis [7]. Other studies have reported an elevation of the leukocyte count and CRP levels among patients with uveitis [24,25]. It is possible that the DS patients that our study recruited had lower levels of such markers, potentially affecting their risks for uveitis. Future research could integrate these tests as part of their analysis to allow for a better understanding of the individual roles these factors may play in the uveitis risk among DS patients. Multimodal imaging techniques, including optical coherence tomography (OCT), optical coherence tomography angiography (OCTA), fluorescein angiography (FFA), and autofluorescence, could also offer a more comprehensive evaluation of the posterior segment of the eye [26]. Our study revealed a notable low number of retinal vasculitis and panuveitis among our DS and non-DS cohorts. Such low incident numbers for these conditions raise the possibility that panuveitis and posterior segment pathologies, such as retinal vasculitis, might have been underdiagnosed or overlooked during routine clinical assessments among pediatric patients. Future studies incorporating multimodal imaging techniques such as optical coherence tomography (OCT), OCT angiography (OCTA), autofluorescence, and fluorescein angiography (FFA) may assist in detecting elusive uveitis cases that might otherwise go undetected in asymptomatic and uncooperative pediatric patients. In summary, by incorporating some of these suggested improvements into future studies, we can better understand the relationship between uveitis occurrence among DS patients.

## Figures and Tables

**Table 1:** Demographic characteristics of Down syndrome and non-Down syndrome.

	Before PSM			After PSM		
	Down Syndrome N = 53,997	Non-Down Syndrome N = 5,445,550	SMD	Down Syndrome N = 53,993	Non-Down Syndrome N = 53,993	SMD
Age	5.21 ± 5.76	6.39 ± 6.03	0.200	5.21 ± 5.76	5.21 ± 5.76	<0.001
Sex						
Female	25,285 (46.83)	2,627,251 (48.25)	0.028	25,283 (46.83)	24,597 (45.56)	0.025
Male	28,406 (52.61)	2,698,624 (49.56)	0.061	28,404 (52.61)	28,401 (52.60)	<0.001
Race						
White	31,459 (58.26)	2,721,257 (49.97)	0.167	31,456 (58.26)	31,462 (58.27)	<0.001
Black or African American	6009 (11.13)	1,042,017 (19.14)	0.225	6009 (11.13)	6006 (11.12)	<0.001
Asian	1651 (3.06)	217,522 (3.99)	0.051	1650 (3.06)	1651 (3.06)	<0.001
Comorbidities						
Other hypothyroidism	1210 (2.24)	8017 (0.15)	0.194	1206 (2.23)	1208 (2.24)	<0.001
Asthma	795 (1.47)	151,927 (2.79)	0.091	795 (1.47)	783 (1.45)	0.002
Congenital malformation of heart, unspecified	440 (0.82)	3927 (0.07)	0.112	437 (0.81)	430 (0.80)	0.001
Hypertensive diseases	316 (0.59)	12,014 (0.22)	0.058	316 (0.59)	316 (0.59)	<0.001
Atopic dermatitis	149 (0.28)	45,580 (0.84)	0.075	149 (0.28)	145 (0.27)	0.001
Celiac disease	70 (0.13)	1792 (0.03)	0.034	69 (0.13)	67 (0.12)	0.001
Crohn’s disease [regional enteritis]	33 (0.06)	1999 (0.04)	0.011	33 (0.06)	35 (0.07)	0.001
Ulcerative colitis	18 (0.03)	1105 (0.02)	0.008	18 (0.03)	18 (0.03)	<0.001

SMD: Standardized mean difference.

**Table 2:** Risk of uveitis exposed to Down syndrome compared to non-Down syndrome.

	Down Syndrome		Non-Down Syndrome		
	N	No. of Event	N	No. of Event	HR (95% CI)
Uveitis	53,993	61	53,993	40	1.33 (0.89–1.99)
H20, Iridocyclitis	53,993	50	53,993	34	1.28 (0.83–1.99)
H30, Chorio-retinal inflammation	53,993	10	53,993	10	1.42 (0.51–4.01)
H35.06, Retinal vasculitis	53,993	10	53,993	0	N/A
H44.11, Panuveitis	53,993	10	53,993	0	N/A
H44.13, Sympathetic uveitis	53,993	10	53,993	10	3.28 (0.36–29.54)

N/A: Not applicable. If the patient’s count is 1–10, the results indicate a count of 10.

**Table 3:** Stratification analysis of the risk of uveitis among different groups.

	Down Syndrome		Non-Down Syndrome		
	N	No. of Event	N	No. of Event	HR (95% CI)
Age					
0–1	23,363	21	23,363	13	1.36 (0.68–2.72)
0–5	32,824	30	32,824	19	1.34 (0.75–2.39)
6–18	21,161	31	21,161	24	1.15 (0.67–1.96)
Sex					
Female	25,278	35	25,278	21	1.49 (0.87–2.56)
Male	28,399	26	28,399	27	0.82 (0.48–1.41)

## Data Availability

The TriNetX platform is a fully deidentified multinational, cloud-based database that adheres to all the relevant standards laid out by Section §164,514(b)(1) of the Health Insurance Portability and Accountability Act (HIPPA), as well as ISO 27001:2013. Due to privacy restrictions, the data from the TriNetX database are not publicly available. However, the population-level aggregate and deidentified data supporting the findings of this study are openly accessible upon reasonable request to the TriNetX administrators through their website (https://trinetx.com/) or by contacting the TriNetX administrators directly (Privacy@TriNetX.com). Alternatively, the corresponding author may also be contacted (doctoraga@gmail.com).

## Supplementary Materials

The following supporting information can be downloaded at https://www.mdpi.com/article/10.3390/medicina60050710/s1: Figure S1: Flow-chart of patient selection; Figure S2: Pictorial representation of the study design; Table S1: Diagnostic codes used for our study’s inclusion, exclusion criteria as well as comorbidities; Table S2: Diagnostic codes used for our study’s outcomes.
