# Supplementary material for: Assessing Uveitis Risk following Pediatric Down Syndrome Diagnosis: A TriNetX Database Study
Source: Medicina (Kaunas). 2024 Apr 25;60(5):710. doi: 10.3390/medicina60050710 (PMC11123068; doi:10.3390/medicina60050710)
Supplement: Supplementary file 1 [file medicina-60-00710-s001.zip › medicina-2961023-supplementary.pdf]

Supplementary Table S1.	Diagnostic codes used for our study’s inclusion, exclusion criteria as well as comorbidities
Supplementary Table S2.	Diagnostic codes used for our study’s outcomes
Supplementary Figure S1	Flow-chart of patient selection
Supplementary Figure S2.	Pictorial representation of the study design

Content	ICD-10-CM
**Inclusion**	
Down syndrome	Q10
**Comorbidities**	
Other hypothyroidism	E03
Asthma	J45
Congenital malformation of heart, unspecified	Q24.9
Hypertensive diseases	I10-I1A
Atopic dermatitis	L20
Celiac disease	K90.0
Crohn's disease [regional enteritis]	K50
Ulcerative colitis	K51
**Exclusion**	
Viral hepatitis	B15–B19
Human immunodeficiency virus	B20, R75, Z21, B97.35
Tuberculosis	A15–A19
Syphilis	A50–A53
Rheumatoid arthritis	M05–M06
Systemic lupus erythematosus	M32
Sjogren's syndrome	M35.0
Rheumatic disease	M31.6, M33.03, M33.13, M33.2, M33.90, M33.93, M34.0, M34.1, M34.9
Behcet's disease	M35.2
Meniere's disease	H81.0
Sarcoidosis	D86
Susac syndrome	I67.7
Juvenile arthritis	M08

Supplementary Table S1. Diagnostic codes used for our study’s inclusion, exclusion criteria as well as comorbidites

Content	ICD-10-CM
Iridocyclitis	H20
Chorio-retinal inflammation	H30
Retinal vasculitis	H35.06
Pan-uveitis	H44.11
Sympathetic uveitis	H44.13

Supplementary Table S2. Diagnostic codes used for our study's outcomes

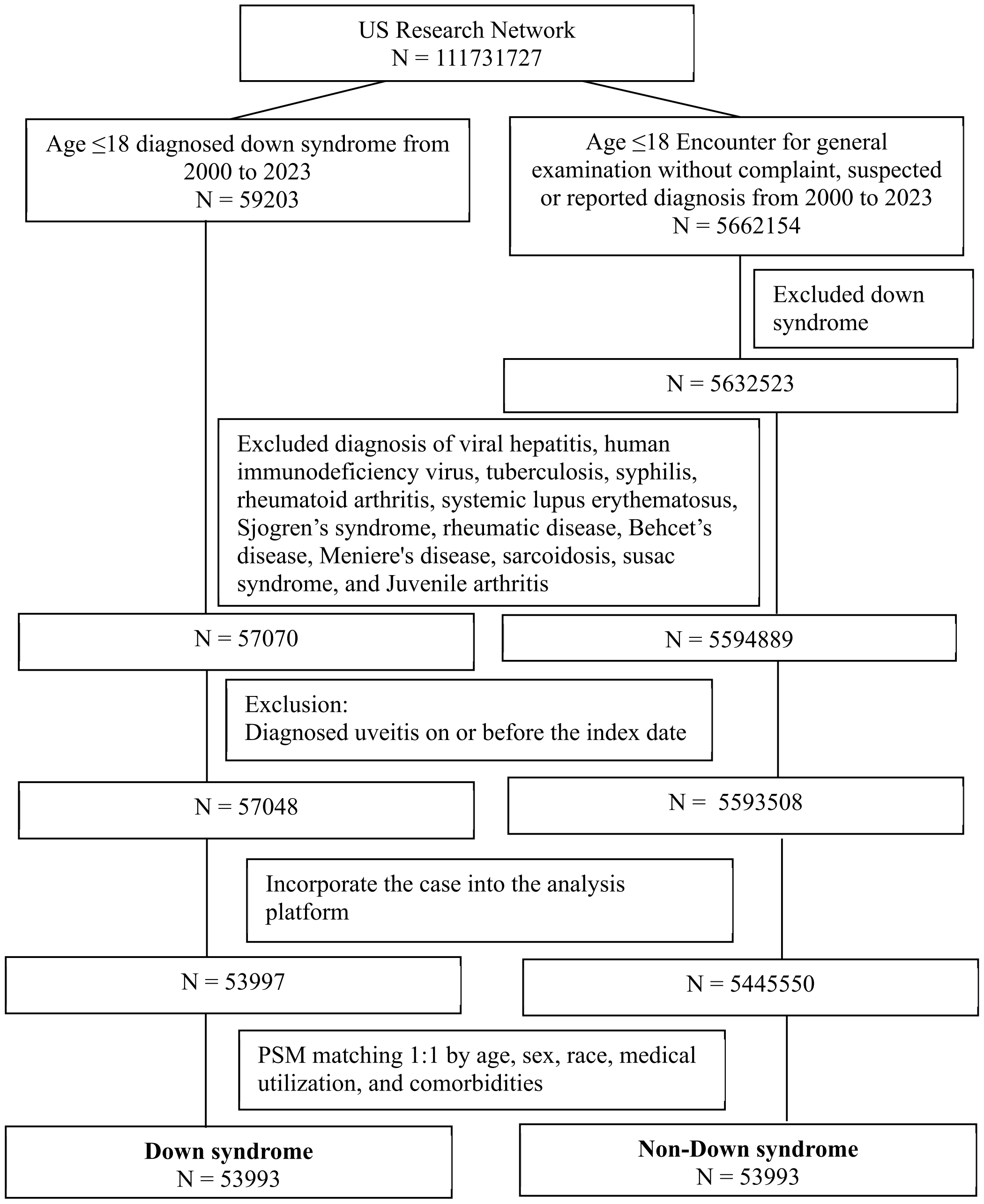
US Research Network
N = 111731727
Age ≤18 diagnosed down syndrome from 2000 to 2023
N = 59203
Age ≤18 Encounter for general examination without complaint, suspected or reported diagnosis from 2000 to 2023
N = 5662154
Excluded down syndrome
N = 5632523
Excluded diagnosis of viral hepatitis, human immunodeficiency virus, tuberculosis, syphilis, rheumatoid arthritis, systemic lupus erythematosus, Sjogren’s syndrome, rheumatic disease, Behcet’s disease, Meniere's disease, sarcoidosis, susac syndrome, and Juvenile arthritis
N = 57070
N = 5594889
Exclusion:
Diagnosed uveitis on or before the index date
N = 57048
N = 5593508
Incorporate the case into the analysis platform
N = 53997
N = 5445550
PSM matching 1:1 by age, sex, race, medical utilization, and comorbidities
Down syndrome
N = 53993
Non-Down syndrome
N = 53993

Supplementary Figure S1 Flow-chart of patient selection

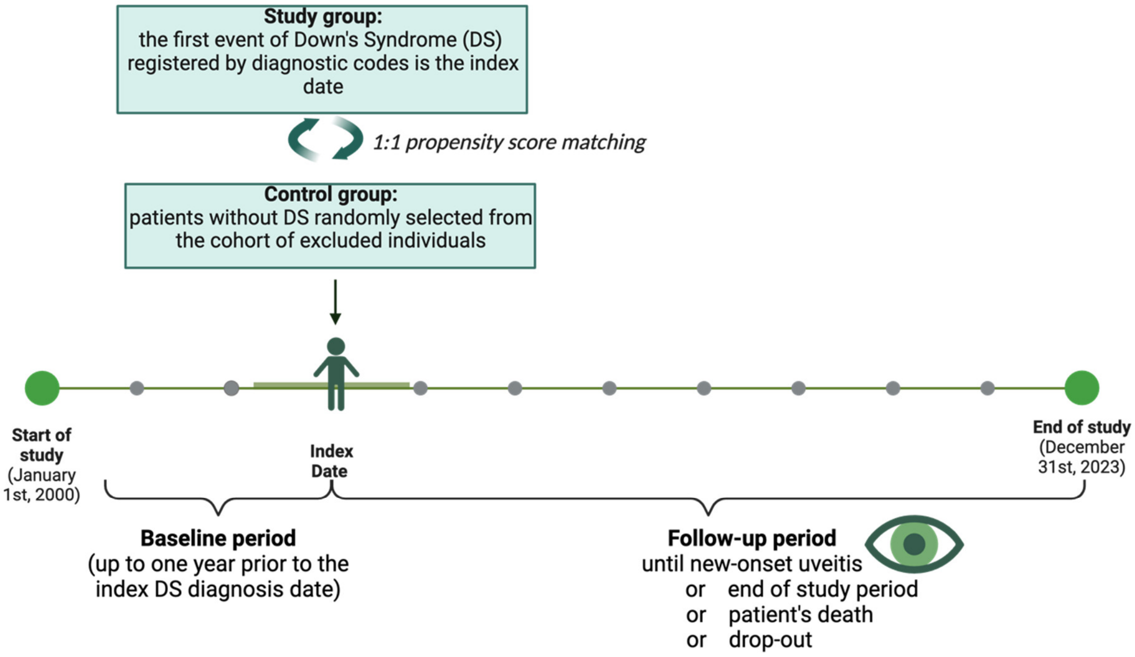

Supplementary Figure S2. Pictorial representation of the study design
